# CHOT LEADERSHIP

**DOI:** 10.1093/geroni/igz038.1546

**Published:** 2019-11-08

**Authors:** Thomas Ferris

**Affiliations:** Texas A&M University, Texas A&M, Texas, United States

## Abstract

The first speaker is Dr. Thomas Ferris the Center Director of the NSF Center for Health Organization Transformation (CHOT). Dr. Ferris will provide a high-level overview of CHOT and provide the audience with a greater understanding of its history, funding, partners, and accomplishments. In this section, the mission, annual research model, and the I/UCRC planning integration framework will be reviewed and discussed. The NSF CHOT is the only Industry-University Cooperative Research Center (I/UCRC) focused on innovations in healthcare delivery in the nation. CHOT researchers work alongside the Industry Advisory Board (IAB) to conduct research that supports the implementation of evidence-based transformational strategies within the healthcare sector. During this symposium, Dr. Ferris will discuss some of the challenges and opportunities that can arise from these partnerships. More importantly, in this section will speak to how value is created through these collaborative efforts and how ultimately this can improve health outcomes.

